# Combining proteogenomics and metaproteomics for deep taxonomic and functional characterization of microbiomes from a non-sequenced host

**DOI:** 10.1038/s41522-020-0133-2

**Published:** 2020-06-05

**Authors:** Duarte Gouveia, Olivier Pible, Karen Culotta, Virginie Jouffret, Olivier Geffard, Arnaud Chaumot, Davide Degli-Esposti, Jean Armengaud

**Affiliations:** 1 Université Paris Saclay, CEA, INRAE, Département Médicaments et Technologies pour la Santé (DMTS), SPI, 30200 Bagnols-sur-Cèze, France; 2 Université de Montpellier, Laboratoire Innovations technologiques pour la Détection et le Diagnostic (Li2D), F-30207 Bagnols sur Cèze, France; 3 INRAE, UR Riverly Laboratoire d’écotoxicologie, centre de Lyon-Villeurbanne, F-69625 Villeurbanne, France

**Keywords:** Next-generation sequencing, Water microbiology, Microbiome

## Abstract

Metaproteomics of gut microbiomes from animal hosts lacking a reference genome is challenging. Here we describe a strategy combining high-resolution metaproteomics and host RNA sequencing (RNA-seq) with generalist database searching to survey the digestive tract of *Gammarus fossarum*, a small crustacean used as a sentinel species in ecotoxicology. This approach provides a deep insight into the full range of biomasses and metabolic activities of the holobiont components, and differentiates between the intestine and hepatopancreatic caecum.

## Introduction

A great diversity of bacteria, archaea, fungi, and viruses (microbiota) inhabit and form complex associations with their animal hosts. In a healthy state, this ensemble of microorganisms and their collective genomes, termed microbiome, can mediate a variety of functions and biological processes in their hosts such as nutrition, energy metabolism, immune responses, or neurotransmission^1^. Microbiome composition and function are sensitive to a variety of biotic, abiotic, and anthropogenic factors, and dysbiosis can have detrimental consequences for the host^2,3^. Among the approaches available for studying microbiomes, metaproteomics—defined as the large-scale identification and quantification of proteins from microbial communities—is rapidly gaining traction as a method to directly observe the protein complement of organisms^4^. This methodology is based on protein shotgun analysis by liquid chromatography tandem mass spectrometry (LC-MS/MS) and interpretation of the MS/MS spectra with available protein sequence databases. The peptide and protein data obtained by metaproteomics can be used for proteotyping the microbial communities present in the sample^5^, estimating biomass contributions^6^, and obtaining a direct understanding of the biochemical functions and interactions of the community^7^.

Due to the paucity of molecular information for numerous animal species such as arthropods, microbial communities from these animals remain poorly characterized. Animal metaproteomics have focused on exploring the microbiomes from large animals such as mammals or birds^8,9^ and studies on small invertebrates are scarce^10,11^. Ideally, metaproteomic data should be interpreted with metagenomics data acquired on the same sample. Similarly, RNA sequencing (RNA-seq) can be used to sequence extracted mRNAs and translate them into putative protein sequences^12^. However, this implies higher costs, time and efforts, and may be challenging when dealing with limited animal resources. The 16S (and/or 18S) rRNA genes of the taxa present can be sequenced and, based on these results, a customized protein database specific to the taxa identified can be proposed. Alternatively, metaproteomic data may be assigned using generalist databases encompassing all known genomes^13^. When the microbial communities of a given host are unknown, assignation of peptide/protein from metaproteomics studies is done only at the highest taxonomical ranks. The absence of a reference genome for the animal host can also hinder the reliable interpretation of the proteomic data, as host peptides that are shared with other taxa can be attributed to non-host proteins present in the database. In any case, the use of metagenomics-derived or multi-organism genome-derived databases enlarges significantly the search space, challenging the search algorithms and compromising the sensitivity of peptide identification^14^. In this context, the use of multi-step database searches helps improve metaproteomics outputs^15,16^.

Here we propose an iterative generalist database search strategy combining high-resolution MS of proteins and RNA-seq of the host to characterize the microbiome of an organism not yet genome-sequenced. In the absence of genomic information on *Gammarus fossarum* from generalist databases, host MS/MS spectra which represent most experimental spectra in the sample could be wrongly attributed. Therefore, we propose to complement generalist databases with an RNA-seq-based protein database specific for the host. Here, we used a previously RNA-seq data set acquired on *G. fossarum*. This data set comprises paired-end Illumina sequences of total mRNAs of specific organs from a large number of male and female gammarids. Reads with at least 90% overlap were assembled into contigs. Contigs were then translated into six reading frames, resulting in a customized protein sequence database (GFOSS) for interpreting MS/MS data (details can be found in ref. ^17^). The workflow was applied for the study of the microbiome of two tissues from the digestive tract of *G. fossarum*—a key species for aquatic systems (body length 2–15 mm) and an emergent model in environmental toxicology^18^. Characterization of the microbiomes associated with the intestine (INT) and hepatopancreatic caeca (HC) is of considerable interest, to better understand their role and possible involvement in mediating the impact of toxicants for the host.

**Fig. 1:**
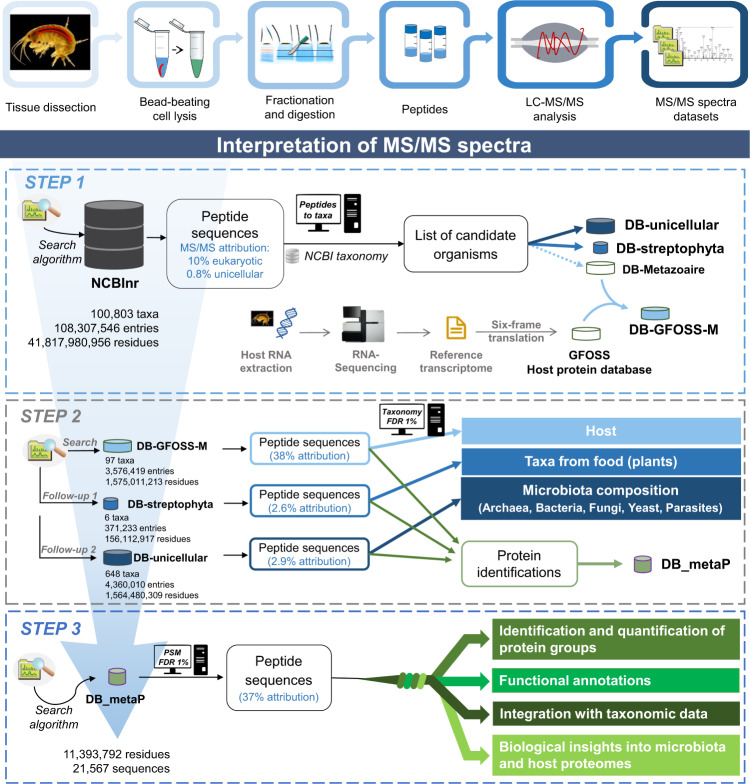
Workflow proposed for the analysis of the gut microbiome of *G. fossarum*. The top panel illustrates the general sample preparation steps and the bottom panel illustrates the multi-step database search and the different types of information retrieved from each round.

Figure 1 schematizes the workflow proposed to analyze the metaproteomes. Three animals were dissected to retrieve the tissues of interest and total protein extraction was performed on individual tissues. As no pooling of tissues was done, each sample informs on the microbiome specific to each individual. The extracted proteins were analyzed twice by tandem MS, leading to datasets totaling 1,044,654 MS/MS spectra. Figure 1 shows how these datasets were interpreted through a multi-step database search. In this bioinformatic pipeline, we tailored a comprehensive database encompassing the molecular information from 100,803 taxa into a reduced sample-specific database.

As a first step, spectra were searched against the generic NCBInr database and 121,378 MS/MS spectra were assigned to peptide sequences (11% attribution rate). Taxon-discriminant peptide sequences highlighted the potential presence of 749 taxa (Supplementary Data 1) whose annotated proteomes were included in three dedicated sub-databases for step 2. In step 2, we performed sequential follow-up searches for each sample to identify the following: (i) host-associated peptides, using DB-GFOSS-M, a database comprising the protein sequences specific to the host organism obtained by RNA-seq^16^, and additional metazoan sequences identified in step1; (ii) food-associated peptides, using DB-streptophyta, which comprises the remaining multicellular eukaryotic proteomes; and (iii) the unicellular microorganism-associated peptides using all eukaryotic and prokaryotic unicellular organisms (DB-unicellular). Most MS/MS signals from the tissues were assigned to the host (38%), the microbiome (3%), and the residual food (<3%; Supplementary Data 2). Approximately 2000 taxon-to-spectrum matches (TSMs) were used for the confident taxonomic identification of the microbiota of the digestive tract (false discovery rate (FDR) < 1%), and quantification of their relative biomasses (Supplementary Data 3). The final database in step 3, DB-metaP, contains all of the protein sequences identified in step 2 for assessing the metabolic activities of both the microbiome and the host. A total of 9532 polypeptides were identified with an FDR of 1% and were grouped into 6466 protein groups (Supplementary Data 4), from which 3717 were attributed to the host organism, 422 to the food, and 1422 to the microbiota.

**Fig. 2:**
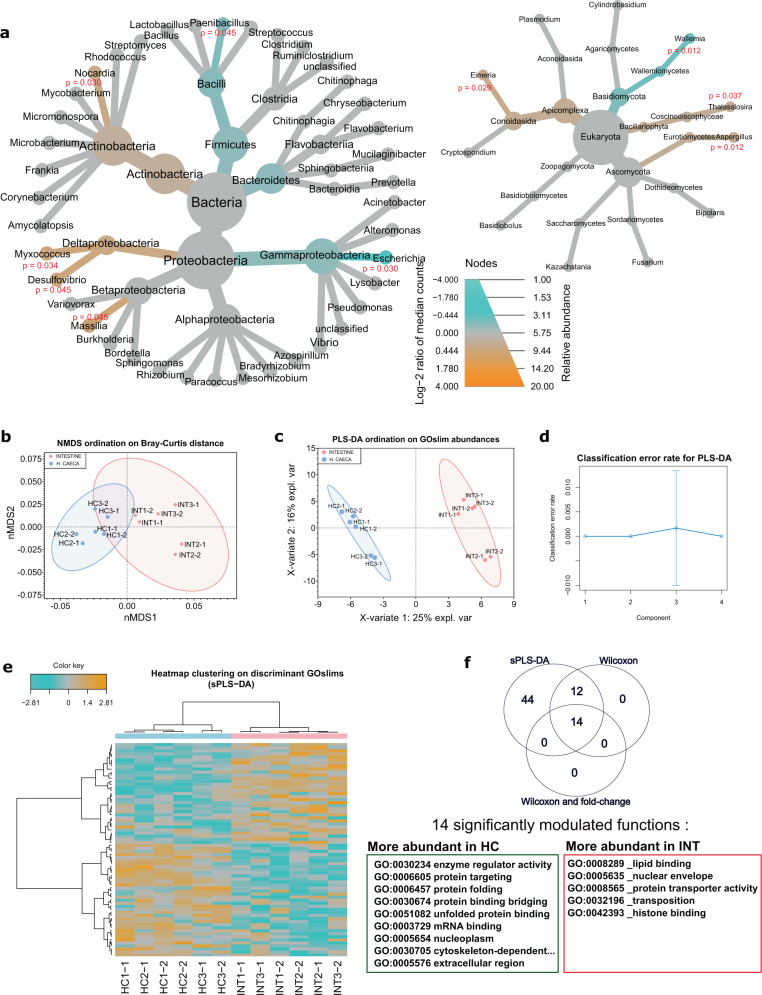
Taxonomical and functional differential analysis of the microbiome. The heat tree in **a** shows the genera that comprise the gut microbiome of G. fossarum. Abundances of each taxon are given by the node size and colored branches mean statistically significant differences in taxa abundance assessed by a Wilcoxon test corrected for multiple comparisons (blue means higher abundance in the intestine and dark orange means higher abundance in the hepatopancreatic caeca). In **b**–**d**, biological (INT1, INT2, INT3, HC1, HC2, and HC3) and technical (represented by the second number of sample label, e.g., INT1_1 and INT1_2) replicates are represented individually. **b** The non-metric multidimensional scaling (NMDS) ordination of Bray–Curtis distances between samples. **c** The partial least-squares discriminant analysis (PLS-DA) ordination of samples according to their Gene Ontology term abundances, with the corresponding classification error rate of the model (**d**). **e** A heatmap clustering of the 70 discriminant features highlighted by sparse PLS-DA analysis. Normal 95% confidence ellipses were calculated for the NMDS and PLS-DA plots. **f** The number of significant features highlighted through univariate analysis.

The microbiome of *G. fossarum* comprises 52 genera: 1 archaeon, 40 bacteria, and 11 eukaryota. Remarkably, we observed that protein biomass contributions were almost equal between bacteria (48%) and unicellular eukaryotes (52%). The most abundant genus was *Streptomyces* with 6% of the assigned signal. Bacteria were dominated by *Proteobacteria* (20%), *Actinobacteria* (16%), *Firmicutes* (9%), and *Bacteroidetes* (4%). All four phyla are commonly found in the gut of other aquatic organisms including supralittoral talitrid amphipods^19^, decapods^20,21^, cladocerans^22^, and fish^23^. Eukaryotic organisms in the gut were dominated by *Ascomycota* (21%), followed by *Apicomplexa* (10%), *Basidiomycota* (10%), *Zoopagomycota* (7%), and *Bacillariophyta* (4%). Figure 2 shows the differential analysis in terms of taxonomy and function when comparing the two tissues from the digestive tract of *G. fossarum*. Figure 2a shows the relative biomass contributions (node size) for the 52 genera. Community composition at the genus level was similar between the two tissues, but some differences in biomass abundance were noted. The differential heat tree highlighted the statistical significance of differences between tissues (colored branches in Fig. 2a). Beta diversity (non-metric multidimensional scaling (NMDS) ordination of Bray–Curtis distances) also indicated a slight dissimilarity between the two organs, despite a higher variability between biological replicates for the INT samples relative to the HC microbiome (Fig. 2b). At the phylum level, we observed that *Actinobacteria* and *Apicomplexa* were more abundant in the HC, whereas *Firmicutes*, *Bacteroidetes*, and *Basidiomycota* were more abundant in the INT. Eight and three genera were more abundant in the HC and the INT, respectively. Among these was the genus *Paenibacillus*, which is notable for the production of chitinase^24^ and could contribute to chitin degradation and recycling during molting of the host. The identification of the genus *Eimeria* (Apicomplexa), more abundantly present in HC, might indicate that these vertebrate parasites use aquatic invertebrates as an intermediate host before infecting fish^25^.

As highlighted in recent literature^26,27^, the functional composition of a microbiome can be more informative than community abundance when characterizing a phenotype. As community abundance in the gut (especially with feces samples) can occasionally change drastically between individuals and species, examining function can help provide a more comprehensive view of host–microbiome interactions. Functional analyses of protein data are represented in Fig. 2c–f. The 1422 protein groups from the microbiome were annotated and their assigned functions were grouped into 148 Gene Ontology subset (GOslim) terms (Supplementary Data 5). These annotations suggested, as expected, that the microbial communities play a role in key functions for the host, as they are dominated by biological processes related to nutrition/digestion (62% of the top 10 biological processes; Supplementary Fig. 1) and, to a less extent, response to stress (7% of the top 10 biological processes; Supplementary Fig. 1). The two most abundant biological processes indicate that the microbiome might have an important contribution to the synthesis of novel biomolecules and to the metabolism of nitrogen compounds. *Streptomyces*, *Mycobacterium*, and *Eimeria* contributed to 31% of the signal associated with these Gene Ontology (GO) terms (Supplementary Data 6). Although both bacteria are known to be implicated in nitrogen metabolism^28^, the data highlight the role of the vertebrate parasite *Eimeria* in metabolizing nitrogen compounds as a source of energy for its life cycle in *G. fossarum*.

The relative abundances of the 148 microbial GOslim functions among samples allowed for a clear separation of both tissues in the partial least-squares discriminant analysis (PLS-DA) plot, suggesting that some functionalities differ in each tissue. A sparse PLS-DA (sPLS-DA) analysis allowed the identification of the subset of GOslims that best discriminate the two tissues (Supplementary Data 7). The heatmap clustering of samples based on these 70 GOslims showed clear distinct profiles of GOslim abundances (Fig. 2e) and the univariate non-parametric analysis highlighted that 14 were significantly modulated between tissues, with a fold change >1.5 and adjusted *p*-value < 0.05 (Fig. 2f and Supplementary Data 7). Interestingly, several GOslims related to enzyme regulation and protein processing/assembly were more abundant in the HC than in the INT (Fig. 2f, GOslims squared in green). The HC are major organs in crustaceans and participate in many metabolic processes such as absorption and storage of biomolecules, detoxification of xenobiotics, and, more importantly, in the secretion of digestive enzymes^29^. The latter function supports the possible microbiome-mediated enzyme regulator activity in this tissue. These functional roles are in line with the results obtained from the host protein analysis. sPLS-DA feature selection from the host proteome highlighted several enzymes that were more abundant in the HC tissue (Supplementary Fig. 2), and that participate in the metabolism of dipeptides and protein macromolecules, synthesis of essential amino-acids, uptake and processing of nutrients, detoxification, and protein transport. These observations suggest that the host and its microbiome cooperate in the processing and regulation of enzymes produced in the HC and in the metabolism of molecules acquired through food. Host proteins more abundant in the INT included some muscle contraction-related proteins (collagen-like proteins and gelsolin), which likely reflect the mechanical role of this tissue for the coating of non-digested products and their later excretion.

The pipeline proposed in this study allows discriminating host, residual food, and microbiome proteins, and identifies the main taxa and their functions at an unprecedented range, including archaea, bacteria, fungi, and parasites. This approach gives more flexibility in database construction, cascade layout, and parameter optimization than currently available tools such as Unipept^5^, MetaLab^15^, MPA^30^, or ProteoClade^31^, which have not been specifically conceived for the analysis of microbiomes from non-sequenced hosts. We believe this strategy can be of great value for future microbiome studies of numerous non-model animals, providing a valid alternative for studies in diverse areas such as environmental biomonitoring, aquaculture, parasitology, or biocontrol. This is especially true for small organisms, for which metagenomics is limited by the small amount of initial sample, or for large cohorts of animals for which metagenomics will be too costly. We capitalized on the rapid advances of protein and RNA-seq technologies to propose an innovative solution in database creation and application for metaproteomics studies of non-sequenced hosts. This workflow can be adapted to a broad range of species and one single sample can provide information on the taxonomic and functional composition of a microbiome, plus host proteome characterization. These approaches pave the way for performing deep mechanistic studies in both host and host-associated microorganisms, and for fuller understanding of microbiome–host relationships.

## Methods

### Sampling and selection of organisms

Gammarids were collected from the Bourbre River in France, acclimatized, and maintained in the laboratory, as previously described^32^, fully compliant with all ethical regulations applicable to animal testing and research in France, i.e., ethical approval is not needed for arthropod studies (decret number 2013-118, Art. R. 214-88, 1). Sexually mature males in amplexus with females in the last stage of their reproductive cycle were selected for this study (before fertilization occurred). The INTs and the HC of three males (i.e., a total of six samples) were aseptically dissected and rapidly frozen in liquid nitrogen for storage at −80 °C.

### Proteome extraction and digestion

Tissues were homogenized in 75 µl of LDS buffer (Invitrogen) containing β-mercaptoethanol. Samples were vortexed, heated for 5 min at 99 °C, incubated for 5 min in an ultrasound bath, and then mechanically homogenized through bead beating in a Precellys® tissue homogenizer (Bertin Technologies). A volume of 30 μL of supernatant was transferred into a new tube and heated for 5 min at 99 °C. Each sample was then subjected to a short SDS-polyacrylamide gel electrophoresis migration at 200 V. The whole protein content from each well was fractionated in three bands per sample, processed, and digested with Trypsin Gold (Promega) using 0.011% ProteaseMAX surfactant (Promega), as described^33^.

### Peptide separation and MS data acquisition

LC-MS/MS analysis was carried out using a data-dependent approach with a Q-Exactive HF mass spectrometer (Thermo Fisher) coupled to an UltiMate 3000 LC system (Dionex-LC Packings) operated as described^34^. Peptides were desalted on a reverse-phase C18 PepMap 100 column. Separation was achieved at a flow rate of 0.2 µL/min and with a 60 min gradient of CH_3_CN, 0.1% HCOOH going from 4% to 25% for 50 min, and from 25% to 40% for 10 min. The gradient full-scan mass spectra were acquired from *m/z* 350 to 1800 with an automatic gain control (AGC) target set at 3 × 10^6^ ions and a resolution of 60,000. The top 20 precursor ions in each scan cycle were subjected to fragmentation through high-energy collisional dissociation. MS/MS scanning was initiated when the AGC target reached 10^5^ ions with a threshold intensity of 17,000 and potential charge states of 2^+^ and 3^+^ after ion selection performed with a dynamic exclusion of 10 s. The experiment was conducted using three biological replicates for each tissue. Peptide solutions from each biological replicate were injected twice to obtain two analytical replicates.

### Taxonomic assignments

MS/MS spectra were assigned to peptide sequences using the MASCOT Daemon 2.6.1 search algorithm (Matrix Science) with the following parameters: trypsin as enzyme, maximum of one missed cleavage, mass tolerances of 5 p.p.m. on the precursor ion and 0.02 Da on the MS/MS, fixed modification of carboxyamidomethylated cysteine (+57.0215), and oxidized methionine (+15.9949) as variable modification. Mascot DAT files were parsed using the ms_peptidesummary function from Matrix Science msparser version 2.5.2. Peptide-to-spectrum matches (PSMs) with the expectation values below 0.05 were validated using the MASCOT homology threshold. Multiple PSMs per MS/MS spectra were allowed in case of ion scores higher than 98% of the top ion score. The peptide sequences for each PSM were mapped to NCBI protein accessions, which were matched to taxa as follows: the files “assembly_summary_refseq.txt” and “assembly_summary_genbank.txt”, downloaded from “ftp://ftp.ncbi.nlm.nih.gov/genomes/ASSEMBLY_REPORTS,” were used to map taxids to RefSeq assemblies (GCF) and GenBank assemblies (GCA). Then, the “GCF/GCA *_assembly_report.txt” files were used to map GCF/GCA to nucleotides and the “*_genomic.gff.gz” files were used to map GCF/GCA to protein accessions. A collation of information was performed following the NCBI taxonomical tree from direct assignation to “canonical” taxonomical levels—species, genus, family, order, class, phylum, and superkingdom. For each taxon at each level, an identification of matching peptide sequences and TSMs was performed, as well as a count of specific or unique peptide sequences, and corresponding specific TSMs. The DB-GFOSS-M, DB-streptophyta, and DB-unicellular databases were built using the proteomes from species identified with (i) more than 9, 7, and 1 specific peptides or (ii) more than 109, 74, and 6 added TSMs for eukaryota, bacteria, and archaea species, respectively. For each species retained, a maximum of ten representative taxa were selected, ordered by the number of TSMs, and with a minimum of two added TSMs.

The FDR for genus identification was calculated after a search against the target database DB-unicellular (#hits_target) and its reversed decoy version (#hits_decoy) and taxonomical assignation of peptide sequence hits. Peptides were assigned to the genus taxonomical rank using the Unipept 4.0 web interface^5^ with default parameters (equate I/L, filter duplicate peptides). The FDR was estimated through comparing the number of taxonomic hits from forward and decoy peptides identified from both databases assuming a threshold of at least three taxon-specific peptides.

### Protein identification and quantification for functional characterization

For step 3, spectra were searched against DB_metaP to identify and quantify all peptides present in the sample. PSMs with an FDR < 1% were filtered and selected for peptide and protein inference. Proteins were grouped if they shared at least one peptide and label-free quantification was based on PSM counts for each protein following the principle of parsimony. Spectral counts from all the proteins in a group were summed for assigning each protein group with an abundance value. Host proteins were analyzed individually, while microbial proteins were grouped in GOslims. Briefly, GO annotations of proteins were performed using a DIAMOND search against the UniRef50 database and the homologs found in this search were mapped to GO terms through the GO annotation database. GOslim terms were obtained by using the Map2Slim option in OWLTools and the generic GOslim list of GO entries. The spectrum count values of all proteins associated with a GOslim term were summed, granting each GOslim term with an abundance value.

### Statistical analyses

Quantitative count values from both taxonomic (number of PSMs) and functional data (spectrum counts) were scaled to their total sum in the sample. The R package *metacoder*^35^ was used for representing taxonomic abundance as a differential heat tree. Univariate differential analysis of taxon and GOslim abundances between conditions was performed through non-parametric Wilcoxon tests corrected for multiple comparisons (Benjamini–Hochberg). The pairwise Bray–Curtis β-diversity indices were calculated using the function vegdist from the vegan package in R. NMDS ordination of Bray–Curtis distances among samples was performed with the function metaMDS. PLS-DA, sPLS-DA, and heatmap clustering were performed with the package mixOmics^36^. The PLS-DA and NMDS plots were drawn with GraphPad Prism v8.0.1 (GraphPad Software).

### Reporting summary

Further information on research design is available in the Nature Research Reporting Summary linked to this article.

## Supplementary information

Supplementary Information

Reporting Summary

Description of Addtional Supplementary Files

Supplementary Data 1

Supplementary Data 2

Supplementary Data 3

Supplementary Data 4

Supplementary Data 5

Supplementary Data 6

Supplementary Data 7

## Data Availability

The mass spectrometry proteomics data have been deposited at the ProteomeXchange Consortium via the PRIDE partner repository with the data set identifier PXD016248.
