# Supplementary material for: Combining proteogenomics and metaproteomics for deep taxonomic and functional characterization of microbiomes from a non-sequenced host
Source: NPJ Biofilms Microbiomes. 2020 Jun 5;6:23. doi: 10.1038/s41522-020-0133-2 (PMC7275042; doi:10.1038/s41522-020-0133-2)
Supplement: Supplementary file 1 — Supplementary Information [file 41522_2020_133_MOESM1_ESM.pdf]

# Supplementary Information

**Combining proteogenomics and metaproteomics for deep taxonomic and functional characterization of microbiomes from non-sequenced hosts**

Duarte Gouveia[1], Olivier Pible[1], Karen Culotta[1], Virginie Jouffret[1], Olivier Geffard[2], Arnaud Chaumot[2], Davide Degli-Esposti[2], Jean Armengaud[1]*

[1] Laboratoire Innovations technologiques pour la Détection et le Diagnostic (Li2D), Service de Pharmacologie et Immunoanalyse (SPI), CEA, INRA, F-30207 Bagnols-sur-Cèze, France.

[2] Irstea, UR Riverly Laboratoire d'écotoxicologie, centre de Lyon-Villeurbanne, F-69625 Villeurbanne, France.

*** Corresponding author**

Jean Armengaud, CEA-Marcoule, DRF-Li2D, Laboratory "Innovative technologies for Detection and Diagnostics", BP 17171, F-30200 Bagnols-sur-Cèze, France;

jean.armengaud@cea.fr; Tel: +00 33 4 66 79 68 02; Fax: +00 33 4 66 79 19 05.

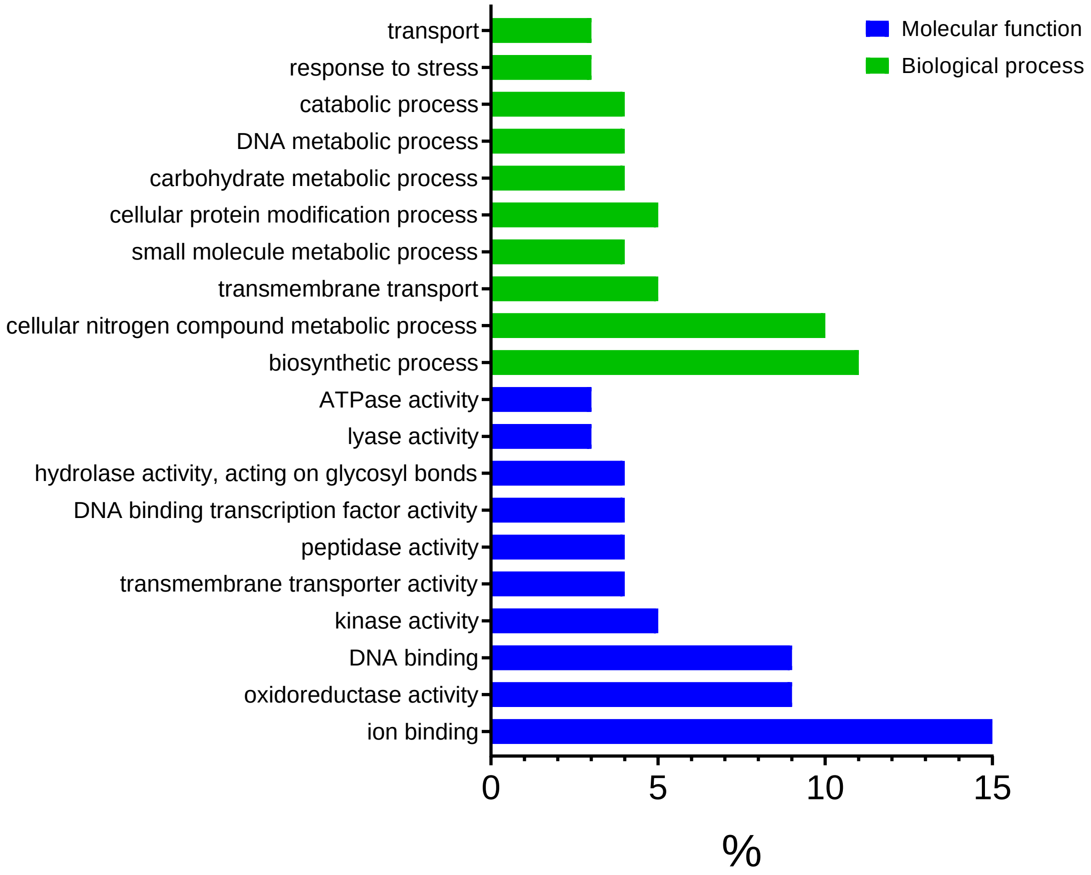


**Supplementary Figure 1** - The ten most abundant GOslim terms for the categories "Molecular Function" and "Biological Process", obtained through annotation of proteins from the microbiota.

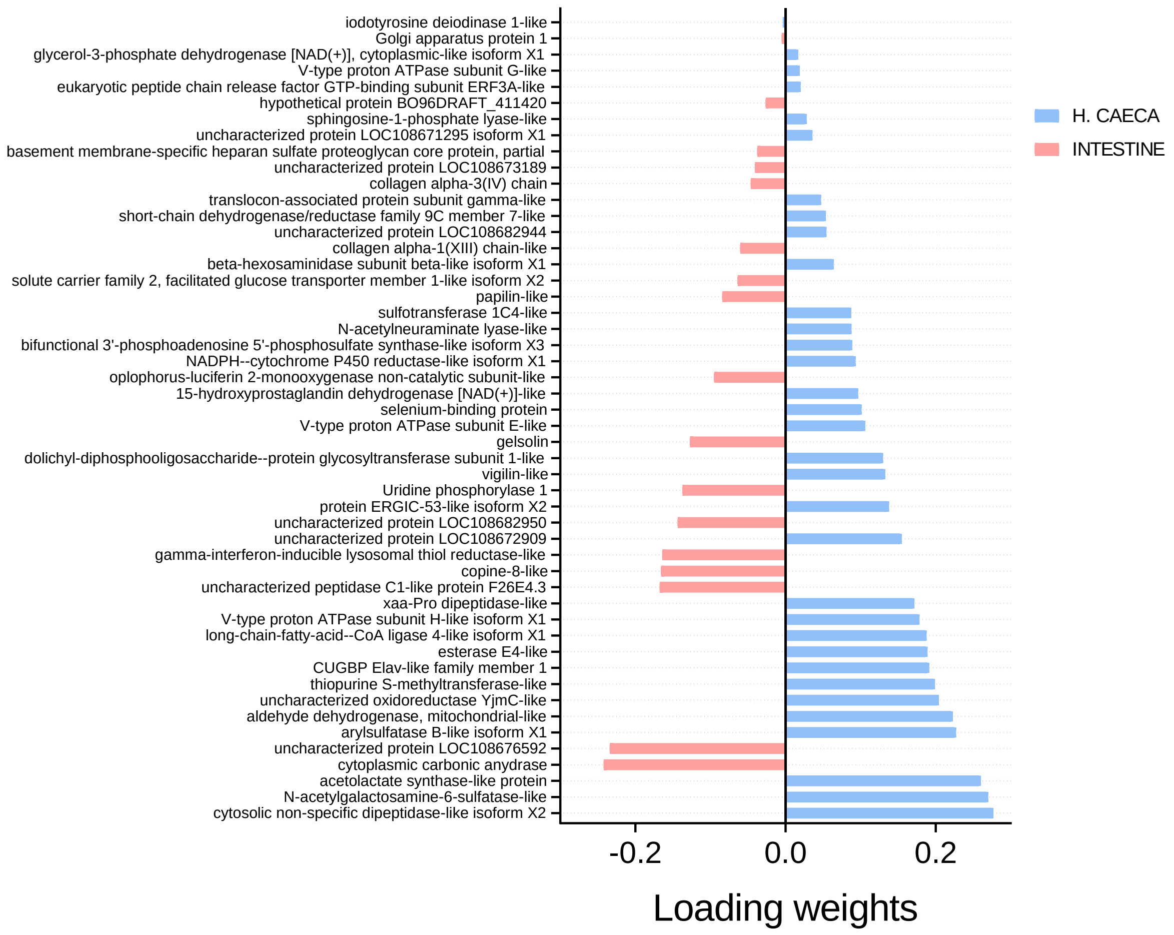


**Supplementary Figure 2 -** Loadings from the sPLS-DA analysis comprising the fifty host protein groups that best discriminate HC from INT.

# Captions for the Supplementary Data Sets

**Supplementary Data 1: Taxa selected in step 1 for the construction of the sub-databases.**

**Supplementary Data 2: Results of database searches for each step of the pipeline.**

**Supplementary Data 3: List of identified genus and their relative abundances based on the number of total and specific TSMs, and number of specific peptides.**

**Supplementary Data 4: List of protein groups identified in Step 3.**

**Supplementary Data 5: List of GOslim annotations and their relative abundances.**

**Supplementary Data 6: List of genus associated with biosynthetic process and cellular nitrogen compound metabolic process**

**Supplementary Data 7: Univariate non-parametric statistics performed on the discriminant features from sPLS-DA.**
