# Supplementary material for: Combining proteogenomics and metaproteomics for deep taxonomic and functional characterization of microbiomes from a non-sequenced host
Source: NPJ Biofilms Microbiomes. 2020 Jun 5;6:23. doi: 10.1038/s41522-020-0133-2 (PMC7275042; doi:10.1038/s41522-020-0133-2)
Supplement: Supplementary file 3 — Description of Addtional Supplementary Files [file 41522_2020_133_MOESM3_ESM.pdf]

Description of Additional Supplementary Files

File: Supplementary Data 1
Description: Taxa selected in step 1 for the construction of the sub-databases.

File: Supplementary Data 2
Description: Results of database searches for each step of the pipeline.

File: Supplementary Data 3
Description: List of identified genus and their relative abundances based on the number of total and specific TSMs, and number of specific peptides.

File: Supplementary Data 4
Description: List of protein groups identified in Step 3.

File: Supplementary Data 5
Description: List of GOslim annotations and their relative abundances.

File: Supplementary Data 6
Description: List of genus associated with biosynthetic process and cellular nitrogen compound metabolic process

File: Supplementary Data 7
Description: Univariate non-parametric statistics performed on the discriminant features from sPLS-DA.
